# Effect of remote ischemic post-conditioning on oxidative stress in blood of STEMI patients treated with primary angioplasty

**DOI:** 10.15171/jcvtr.2016.24

**Published:** 2016-09-30

**Authors:** Hassanali Lotfollahi, Mustafa Mohammadi, Samad Ghaffari, Reza Badalzadeh, Bahram Sohrabi, Naser Aslanabadi, Ahmad Separham, Ali Golmohammadi, Ali Abbasnejad, Mehri Roshani

**Affiliations:** ^1^Cardiovascular Research Center, Tabriz University of Medical Science, Tabriz, Iran; ^2^Department of Physiology, Faculty of Medicine, Tabriz University of Medical Science, Tabriz, Iran

**Keywords:** STEMI, Remote Ischemic Post-conditioning, Primary Angioplasty, Oxidative Stress, MDA

## Abstract

***Introduction:*** This study designed to use remote ischemic post conditioning (RIPC) as a protective strategy during percutaneous coronary intervention (PCI) in patients with ST segment elevation myocardial infarction (STEMI) to reduce myocardial cells damage due to reperfusion injury.

***Methods:*** Sixty-one patients were divided into test group (32 patients) receiving RIPC and control group (29 patients). Patients were included with first MI who had 20-80 years old. The RIPC protocol was applied on patients arm in three successive episodes during the opening of infarct-related artery (IRA). Whole blood sample were taken from patients after the first episode before IRA opening and after the third episode after IRA opening. The serums were extracted and stored in the freezer -70˚C to determine the levels of glutathione peroxidase (GPX), superoxide dismutase (SOD), total antioxidant capacity (TAC) and malondialdehyde (MDA).

*** Results:*** The levels of GPX and SOD after the first episode of RIPC were significantly higher in test group than control group (*P* < 0.001). Similar alterations of these enzymes were obtained after IRA opening (after third episode). In addition, the levels of TAC remained unchanged in control patients but it was significantly increased after the third episode of RIPC in test patients (*P* < 0.001). Finally, the MDA level was increased in control group in comparison with test group, and administration of RIPC in test group prevented the enhancement of MDA levels significantly (*P* < 0.001).

***Conclusion: ***The results indicated that RIPC protocol has protective properties in patients with STEMI through enhancing the antioxidant potentials and decreasing lipid peroxidation.

## Introduction

ST segment elevation myocardial infarction (STEMI) is one of the leading causes of mortality and morbidity worldwide. Infarct size is the main determinant of prognosis.^1^ The protective role of ischemic post-conditioning has been showed based on the observation that a slow or intermittent reperfusion rather than abrupt reperfusion reduces the damage caused by ischemia-reperfusion.^2^ Remote ischemic post-conditioning applied not in the heart but in distant organs such as leg or arm can release post-conditioning mediators that affect the myocardial flow.^2^ Post-conditioning mechanisms are not exactly known but likely include numerous mediators and triggers.^3^ Acute STEMI is a dynamic or static phenomenon, so the therapeutic maneuvers to protect myocardial tissue at risk of no reflow situation can be effective.^4^ Reduction of infarct size is a main goal of treatment and can be achieved efficiently with primary angioplasty.^5^ Successful and timely reperfusion with primary angioplasty or primary percutaneous coronary intervention (PPCI) results in reduction of infarct size, preservation of ventricular function and improving of clinical outcomes.^1^ However, the sudden establishment of blood flow causes fatal myocardial cells injuries, which may limit the therapeutic benefit.^1^ Supplementary mechanical treatments such as thrombectomy and distal protection devices have short-term and temporary benefits. One of the best alternatives for treatment and protection of heart cells is using of the inherent mechanisms.^5^

Remote ischemic preconditioning has recently been shown to effectively attenuate myocardial ischemia/reperfusion injury in patients,^6,7^ but the underlying mechanisms are incompletely understood.^8^ It has been reported that following reperfusion in ischemic arteries, the free radical formation levels increased that can be very risky for heart tissue.^9^ Percutaneous coronary intervention (PCI), through imposing the effects of ischemia-reperfusion, is associated with increased oxidative stress.^10^ It is known that if short episodes of ischemia are exerted on limb, before the main ischemia to the myocardium, it will lead to the lessening of the extent of myocardial injury efficiently.^5^

Oxidative stress, an imbalance between the production of free radicals and antioxidant defense systems of the body, is strongly associated with cardiovascular disease and its complications.^11^ It was shown during cardiovascular diseases, oxidative stress increased,^12^ and treatment with antioxidants had beneficial effects.^13^ Loeper and colleagues have suggested the increased lipid peroxidation and protective enzymes such as superoxide dismutase (SOD) in MI and unstable angina.^14^ Previous studies have shown that after successful reperfusion, the levels of malondialdehyde (MDA) enhanced and antioxidants such as vitamin C, SOD and glutathione peroxidase (GPX) were declined.^15^ Also it has been reported that after thrombolytic therapy and PCI levels of MDA increased.^15-18^

Considering the importance of the issue and because the protective effects of RIPC protocol has been known in some conditions previously, the aim of this study was to use this protocol as a protective strategy during primary PCI in patients with STEMI to reduce myocardial oxidative stress and cell damages due to reperfusion injury.

## Materials and Methods

### Patients

**Figure 1:**
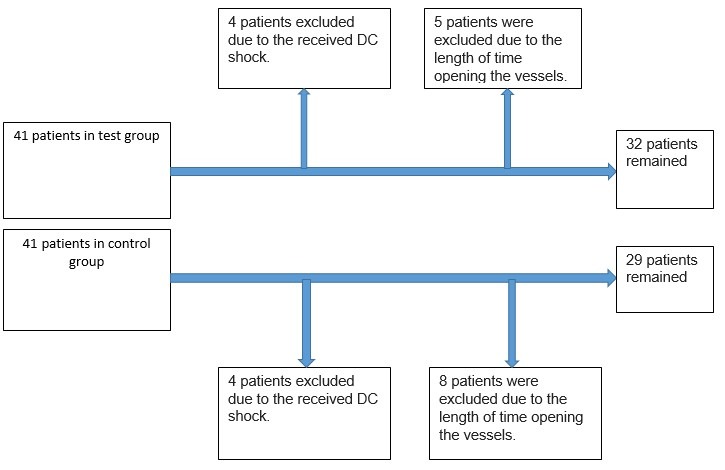
Study flowchart.

This study was conducted on patients with first STEMI undergoing PPCI that were referred to the emergency department of Madani Heart Center of Tabriz University of Medical Sciences during days and nights. Sixty-one patients including 32 patients as test group, 29 patients as control group were enrolled to the study (Figure 1). Allocated patients in this investigation were who with first MI (20-80 years) in the first 12 hours after beginning of symptoms and without prohibitions for thrombolytic therapy. Patients with left bundle branch block (LBBB), pacemaker, cardiogenic shock or DC shock receipt, patients undergoing rescue PCI or candidate for emergency CABG were excluded from the study. Basic characteristics of patients, including epidemiological data, distribution of risk factors and treatments were considered in analysis.

### Determining the sample size

The sample size of the study was calculated using a related software (PS software for power and sample size calculations), with considering the significance level set at 0.05 and power of study set as higher than 80%, based on the results of previous study.^13^ The resultant sample size was 36 patients for each group; however, for increasing the accuracy of this experiment, the sample size per group was increased to 41 patients.

### Remote ischemic post-conditioning protocol

**Figure 2:**
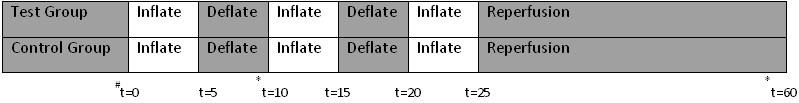
Timeline protocol in the test and control groups. In inflate times (at t = 0, 10, and 20 minutes) the cuff was inflated 30 mm Hg above the systolic pressure in the test group and 30 mm Hg below the diastolic pressure in the control group. At deflate times (t = 5, 15, and 25 minutes) the cuff was deflated in both groups.

RIPC protocol began immediately after diagnostic angiography and deciding to start PPCI. RIPC protocol was performed by inflation and deflation in three episodes which each takes 5 minutes, intermittently (Figure 2). This protocol was applied in the first episode of inflation before the opening of infarct related artery (IRA) and the two next episodes performed after opening of artery.

According to this protocol in the test group the cuff (in the arm without IV-line) was inflated for 5 minutes to 30 mm Hg higher than the patient’s systolic pressure and then deflated for 5 minutes.^5^

In control group cuff pumped 30 mm Hg below the patient’s diastolic pressure and then deflated for 5 minutes. In both groups the first balloon inflation and deflation was conducted during the crossing of guide-wire from the lesion in the early 10 minutes of angioplasty. Two next episodes of RIPC were repeated after the opening of the IRA.^5^

### Preparing blood sample

First blood sample was collected immediately after opening of infarcted related artery (after the first episode of post-conditioning). Also second blood samples were collected one hour after the start of application of the protocol (50 minutes after opening the IRA). Whole blood sample were taken and the serums were extracted and then stored in the freezer -70 to determine the levels of GPX, SOD, TAC and MDA.

### Determination of antioxidant enzymes

### Superoxide dismutase

For the quantitative in vitro determination of SOD in whole blood, SOD activity was determined using a RASOD laboratory kit (Randox Crumlin, UK) according to Delmas-Beauvieux. SOD activity was measured at 505 nm by a spectrophotometer (Pharmacia Biotech; England).^19^ This method employs xanthine and xanthine oxidase (XOD) to generate superoxide radicals which react with 2-(4-iodophenyl)-3-(4-nitrophenol)-5-phenyltetrazolium chloride (I.N.T.) to form a red formazan dye. The SOD activity is then measured by the degree of inhibition of this reaction. One unit of SOD is that which causes a 50% inhibition of the rate of reduction of INT under the conditions of the assay. SOD units/mL of whole blood equal SOD units/mL from standard curve in dilution factor. To Converting SOD (units/g hemoglobin), SOD units/mL was divided into g (hemoglobin/mL).

### Glutathione peroxidase

GPX activity was determined using a RANSEL laboratory kit (Randox Crumlin, UK) according to the method of Paglia and Valentine. GPX catalyses the oxidation of glutathione (at a concentration of 4 mmol/L) by cumene hydroperoxide.^20^ In the presence of glutathione reductase (at a concentration ≥ 0.5 units/L) and 0.28 mmol/L of NADPH, oxidized glutathione is immediately converted to the reduced form with concomitant oxidation of NADPH to NAD+. The decrease in absorbance at 340 nm (37°C) was measured using a spectrophotometer (Pharmacia Biotech, England), and then GPX concentration was calculated from the following formula: GPX U/L of sample = 8412 × ΔA 340 nm/min ΔA = difference of blank value from sample value GPX U/mg protein = GPX U/mL/protein concentration/mL.

### Total antioxidant capacity

Serum total antioxidant capacity (TAC) was determined for a quantitative assessment of in vivo antioxidant status using a commercially available kit (Randox) based on the trolox equivalent of antioxidant capacity according to the manufacturer’s instructions.^21^

### Malondialdehyde assessment

MDA, the final product of lipid peroxidation, was measured in the blood samples based on Esterbauer and Cheeseman method, MDA responses to thiobarbituric acid and produces a pink pigment that has a maximum absorption at 532 nm.^22^

### Statistical analysis

The data were analyzed using the SPSS version 22, and all values were expressed as means ± standard error (SE) of the means. After the initial analysis of the variables, the data was subjected to analysis with repeated measures define factors test. Differences were considered statistically significant when *P < *0.05.

## Results

The basic characteristics of 61 patients with acute myocardial infarction that referred to Madani Heart Center and treated with PPCI are listed in Table 1. Statistical analysis shows no significant differences among data.

**Table 1:** The basic characteristics, history and medical records of patients in experimental groups

**Basic parameter**	**Test group**	**Control group**	***P *** ** value**
Male, n (%)	25 (78.10)	23 (79.30)	0.7
Female, n (%)	7 (21.90)	6 (20.70)	0.7
Age (mean ± SEM)	58 ± 13	61 ± 11	0.38
BMI (mean ± SEM)	24 ± 1	25 ± 2	0.4
History, n (%)			
Family history of MI	6 (18.80)	5 (17.20)	0.93
Smoking	11 (34.40)	8 (27.60)	0.63
Hyperlipidemia	8 (25)	3 (10)	0.15
Hypertension	15 (46.90)	11 (37.90)	0.56
Diabetes mellitus II	6 (18.80)	8 (27.60)	0.38
Unstable angina	9 (28.10)	6 (20.70)	0.5
Previous angioplasty	1 (3.10)	0 (0)	0.32
Aspirin intake	4 (12.50)	4 (12.5)	0.88
Insulin	2 (6.30)	0 (0)	0.16
Metformin	5 (15.60)	3 (10.30)	0.54
Glibenclamide	5 (15.60)	3 (10.30)	0.54
Beta-blocker	7 (21.90)	7 (6.90)	0.09
Statins	4 (12.50)	1 (3.40)	0.19
Dynamic characteristics, mean ± SEM			
LVEF	39.53± 9.1	38.62± 9.9	0.7
Time to PCI initiation (h)	5.5±‏ 3.65	4.98±‏ 2.96	0.6
Heart rate	83±‏ 16	78±‏ 14	0.28
Systolic pressure	143±‏ 35	142±‏ 30	0.95
Diastolic pressure	79±‏ 11	78±‏ 10	0.69

Abbreviations: BMI, body mass index; LVEF, left ventricular ejection fraction. Results are expressed as number of cases, percentage, mean ± SEM, for n=32 in test group and n=29 in control group. Statistical analysis (independent samples test) indicated no significant differences among data (*P < *0.05).

Biochemical parameters before PCI are listed in Table 2. Statistical analysis (independent samples test) indicated no significant differences among data (*P < *0.05).

**Table 2:** Biochemical parameters before PCI

**Biochemical parameters**	**Control group**	**Test group**	***P *** ** value**
Hemoglobin (g/dL)	14.6± 1.7	14.3± 1.8	0.52
Hematocrit (%)	42.8± 4.6	42.8± 4.6	0.32
Ccreatinine (mg/dL)	1.08± 0.17	1.17± 0.3	0.17
Blood sugar (mg/dL)	180± 106	151± 51	0.17
Urea (mg/dL)	15.39± 3.5	16.6± 6.3	0.35
Total cholesterol (mg/dL)	173± 40	163± 35	0.37
Triglyceride (mg/dL)	123± 53	139± 107	0.51
HDL (mg/dL)	38± 9	37± 18	0.81
LDL (mg/dL)	101.75± 8.2	105.15± 9.2	0.78

Results are expressed as mean ± SEM, for n=32 in test group and n=29 in control group. Abbreviations: HDL, high-density lipoprotein; LDL, low-density lipoprotein.

**Figure 3:**
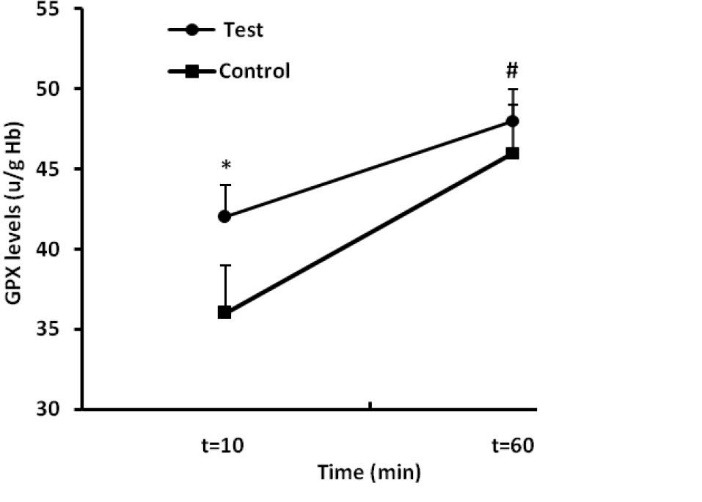
Comparison of GPX levels alterations after first episode of RIPC (at t=10 min, first sample) and at the end of RIPC (at t=60 min, second sample) between experimental groups. * P < 0.001 as compared with first sample (t=10) of control group; # P < 0.01 as compared with second sample (t=60) of control group.

In addition, the results of this study showed that GPX levels were significantly increased in test group compared to the control group (Figure 3). Comparison between two groups showed that the GPX levels after first episodes of RIPC (first sample) in the test group was higher than those of control group (*P < *0.001). Furthermore, the GPX levels after the end of RIPC (second sample) had similar alterations, so that its level in test group was higher than controls (*P < *0.01; Figure 3).

**Figure 4:**
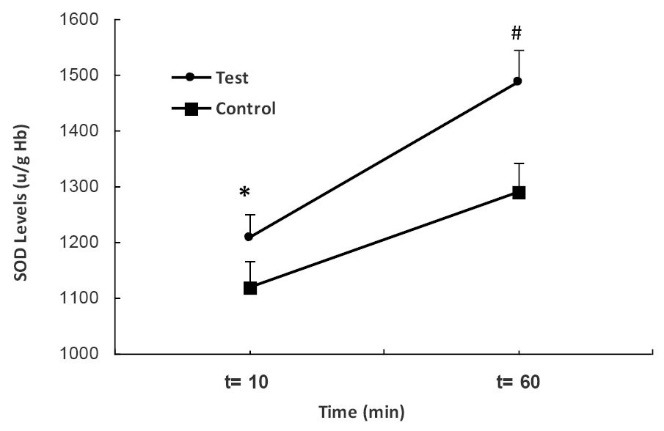
Comparison of SOD levels alterations after first episode of RIPC (at t=10 min, first sample) and at the end of RIPC (at t=60 min, second sample) between experimental groups. * P < 0.001 as compared with first sample (t=10) of control group; # P < 0.01 as compared with second sample (t=60) of control group.

The SOD levels in the test group were increased significantly as compared to the control group (Figure 4). As indicated in Figure 4, the SOD levels of first period in the test group was higher than those of control group (*P < *0.001). Additionally, SOD levels in the second sample of test group were enhanced significantly in comparison with control group (*P < *0.01).

**Figure 5:**
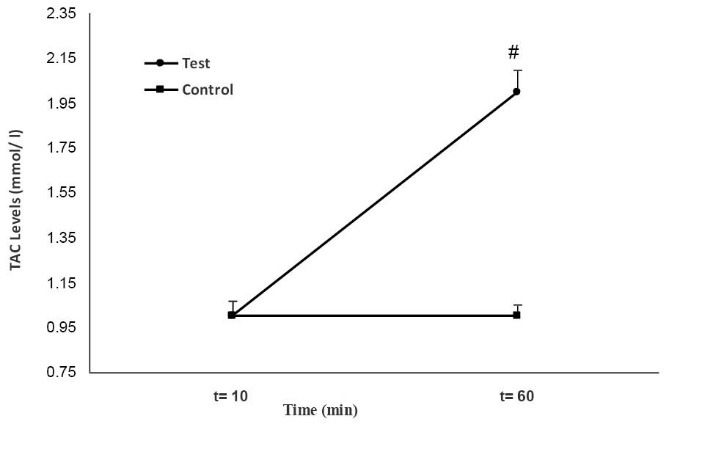
Comparison of total antioxidant capacity (TAC) levels alterations after first episode of RIPC (at t=10 min, first sample) and at the end of RIPC (at t=60 min, second sample) between experimental groups. *P < 0.001 as compared with second sample (t=60) of control group.

As indicated in Figure 5, at the first episode of RIPC (at t=10 minutes) the TAC levels in the test group was similar to the control group. However, the levels of TAC in the test group at the end of RIPC (second sample) were significantly increased in comparison to the control group (*P < *0.001) (Figure 5).

**Figure 6:**
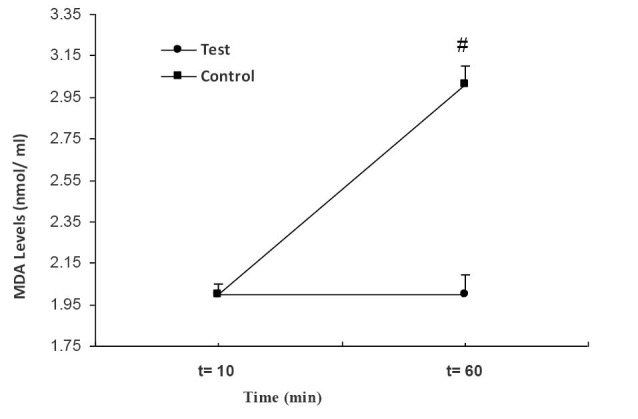
Comparison of MDA levels alterations after first episode of RIPC (at t=10 min, first sample) and at the end of RIPC (at t=60 min, second sample) between experimental groups. *P < .001 as compared with second sample (t=60) of control group.

Finally, the levels of MDA in the test group remained unchanged in the first and second samples of blood, indicating the preventive influence of RIPC protocol on MDA levels (Figure 6). On the other hand, MDA levels in the second sample of control group was significantly higher than those of the first sample in that group (*P < *0.002).

## Discussion

This study aimed to investigate the effect of RIPC in patients with acute myocardial infarction treated with primary angioplasty. Our results indicated the significant increase in the antioxidant defense system (SOD, GPX) and TAC levels following the application of RIPC.

In consistent with our finding, it was shown that the remote ischemic postcondioning enhanced the GPX activities in heart, brain, intestinal and kidney tissues.^23^ GPX protects the cellular and sub-cellular membranes against the oxidative stress injury by removing of lipid peroxides and hydrogen. Increased activity of this enzyme lead to reducing the damages produced by the enhancement of lipid peroxidation that may act by counteracting the harmful products.^24,25^ The levels of GPX in the test group were higher than that of the control group which could be due to a use of protocol RIPC.

SOD is also one of the most important antioxidant mechanisms against reactive oxygen species. It accelerates to detoxify the toxic superoxide radical (O2-) that is produced during oxidative energy processes and convert them to molecular oxygen and hydrogen peroxide.^26^ Administration of RIPC in test group led to increased levels of SOD.

Evaluation of the TAC gives more biological relevant information than that of the individual levels of specific antioxidants. TAC levels consider the cumulative effect of all antioxidants present in plasma and it is used for evaluating the effect of several physiological conditions on plasma in human and animals. It has been suggested that estimation of TAC may be a useful parameter for assessment of oxidative stress.^27^ TAC was higher in the test group compared to the control group that may protect cells from oxidative stress and consistent with previous reports.^28,29^ Thus, this finding also indicates the protective influence of RIPC in PCI setting.

MDA is derived from oxidative destruction of lipids in cell membranes, and the alteration in MDA concentration can be an indicator of lipid peroxidation and oxidative cell injury.^30-32^ It has been reported that the amount of lipid peroxidation depends on the cell injury. In the control group, MDA level increased significantly, which is consistent with previous studies.^15^ But, MDA level remained unchanged in RIPC-receiving group in comparison with its baseline value, indicating that RIPC protocol may prevent the over production of MDA and thereby reduce the levels of oxidative stress. Therefore, it can be concluded that RIPC in STEMI patients treated with PPCI could prevent the extension of cell damages caused by reperfusion injury.

In conclusion, our data showed the antioxidant variables in the experimental group were increased significantly compared to the control group and MDA levels was reduced significantly. It can be suggested that high levels of endogenous antioxidants (GPX, SOD and TAC) can indicate favorable effects of RIPC in patients with acute myocardial infarction undergoing PPCI.

## Limitations of the study

Regarding the limitations of this study and results of previous studies, we did not measure circulating free radicals and measured oxidative stress.

## Ethical Approval

The study protocol was approved by the ethics committee of Tabriz University of Medical Sciences (no: 93109; 06/10/2014). Written consents were obtained from all patients prior to inclusion in the study.

## Competing interests

Authors declare no conflict of interest in this study.

## Acknowledgments

We wish to thank Cardiovascular Research Center of Tabriz University of Medical Sciences for it financially support of our study.
